# Neddylation, an Emerging Mechanism Regulating Cardiac Development and Function

**DOI:** 10.3389/fphys.2020.612927

**Published:** 2020-12-17

**Authors:** Jie Li, Jianqiu Zou, Rodney Littlejohn, Jinbao Liu, Huabo Su

**Affiliations:** ^1^Vascular Biology Center, Medical College of Georgia, Augusta University, Augusta, GA, United States; ^2^Protein Modification and Degradation Lab, School of Basic Medical Sciences, Guangzhou Medical University, Guangzhou, China

**Keywords:** NEDD8 (neural precursor cell expressed developmentally down-regulated 8), Ubiquitin-like protein, Neddylation, cardiomyopathy, Heart Failure

## Abstract

Defects in protein quality control have been increasingly recognized as pathogenic factors in the development of heart failure, a persistent devastating disease lacking efficacious therapies. Ubiquitin and ubiquitin-like proteins, a family of post-translational modifying polypeptides, play important roles in controlling protein quality by maintaining the stability and functional diversity of the proteome. NEDD8 (neural precursor cell expressed, developmentally downregulated 8), a small ubiquitin-like protein, was discovered two decades ago but until recently the biological significance of NEDD8 modifications (neddylation) in the heart has not been appreciated. In this review, we summarize the current knowledge of the biology of neddylation, highlighting several mechanisms by which neddylation regulates the function of its downstream targets, and discuss the expanding roles for neddylation in cardiac physiology and disease, with an emphasis on cardiac protein quality control. Finally, we outline challenges linked to the study of neddylation in health and disease.

## Introduction

Heart failure is expected to remain the main cardiovascular event responsible for hospitalization throughout the world, including the US (Benjamin et al., 2019). Substantial research efforts in recent decades have identified derangement of cellular protein homeostasis as an important mechanism underlying the initiation and progression of different forms of cardiac disease. These findings have led to heightened interest in protein quality control in cardiomyocytes. In general, protein quality is closely monitored by protein chaperones and degradation machineries. In response to cellular stresses that lead to protein misfolding and damage, chaperones first attempt to restore the tertiary conformation of the protein. The proteasome and (macro) autophagy subsequently serve to degrade proteins that escape the surveillance of chaperones as well as those that are no longer needed by the cell.

Although these key factors have long attracted considerable research attention, an emerging concept posits that diverse post-translational modifications (PTMs) also play pivotal roles in protein quality control and homeostasis. Upon synthesis, nascent proteins can be modified by chemical groups or polypeptides via enzymatic reactions. In eukaryotic cells, more than 300 PTMs have been identified, including acetylation, methylation, phosphorylation, glycosylation, ubiquitination and sumoylation, among many others. PTMs alter the stability, subcellular distribution, activity and interactome of the modified substrates, and therefore have pleiotropic impacts on the functionality of target proteins. These PTMs represent vital mechanisms that regulate virtually every aspect of cell physiology. Not surprisingly, flaws in these processes can result in many forms of human disease, including heart failure.

The ubiquitin superfamily—the major polypeptide class of protein modifiers—comprises ∼17 members, including ubiquitin (Ub), SUMO proteins, NEDD8, ISG15, FAT10, HUB1, UFM1, URM1, and ATG8 (Li et al., 2018). Ubiquitin-like proteins (UBLs) share varying degrees of similarity in amino acid sequence and tertiary structure with ubiquitin. UBLs are often synthesized as precursor proteins that require proteolytic cleavage and maturation, and employ an E1-E2-E3 enzymatic cascade similar to that of ubiquitination for attachment to protein substrates (Sun, 2003). Despite these broad similarities between Ub and UBLs, individual UBLs are functionally distinct from Ub, and indeed from each other, and have broad biological functions (Gomes, 2018).

Polypeptide PTMs have several unique features compared with chemical modifications. First, addition of one or a chain of polypeptides to proteins can significantly alter the molecular weight and charge of proteins and thereby drastically impact their structure and function. Second, these modifications are catalyzed by a hierarchy of enzymes that are much more complex than those that mediate chemical modifications. In the case of ubiquitination, there are several E1 activating enzymes, a dozen E2 conjugating enzymes and hundreds of E3 ligases (Glickman and Ciechanover, 2002), which coordinately mediate the modification of thousands of protein substrates. Notably, there are approximately 100 deubiquitinases that reverse this modification. Third, proteins can be modified at one or multiple lysine residues by either a single molecule (mono- and multi-mono modification) or a chain of modifiers. Since each individual polypeptide modifier contains multiple lysine residues that can serve as attachment points for chain extension, protein substrates can be modified by homo- and heterotypic polymers, and even branched polymers (Glickman and Ciechanover, 2002), further expanding the diversity of modification patterns. Together, the complexity of the enzymatic cascade and modification linkage types confer multiple points for regulation of protein function and provide countless opportunities for propagating specific signals.

**FIGURE 1:**
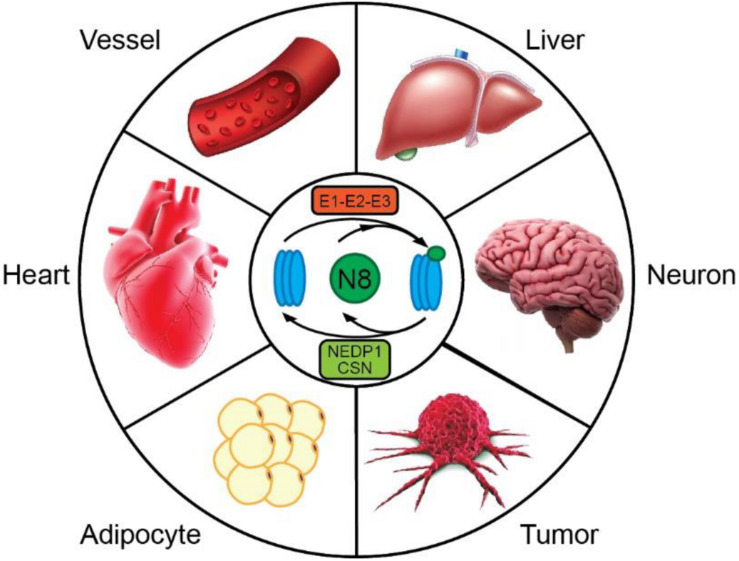
Neddylation has emerged as a powerful mechanism for regulating cellular physiology in a variety of tissues. Dysregulation of neddylation has been linked to vascular diseases, such as neointima formation and atherosclerosis, liver steatosis, cognitive deficits, tumor growth, obesity, and heart failure. Modulation of this pathway may provide opportunities for combating diverse human disorders.

In the heart, ubiquitin, SUMO and ISG15 PTMs have been linked to the pathogenesis of cardiomyopathy (Wang and Robbins, 2006; Kho et al., 2011; Rahnefeld et al., 2014; Willis et al., 2014; Mendler et al., 2016) and have become attractive therapeutic targets in the prevention and treatment of cardiac disease (Henning and Brundel, 2017). In contrast, the significance of other UBLs remains rarely explored in cardiac tissues. Among all UBLs, NEDD8 (neural precursor cell expressed developmentally downregulated 8) shares the highest homology with Ub, exhibiting approximately 60% sequence identity, and like Ub, contains three β-pleated sheet structures centered around one central α helix (Rao-Naik et al., 1998). In the last decade, the conjugation of proteins with NEDD8, hereafter termed neddylation, has emerged as a novel regulatory mechanism for the control of diverse cellular functions. Moreover, recent studies have pointed to the NEDD8 system as a major contributor to tissue homeostasis and as a key player in the progression of different disease states, including developmental defects (Lykke-Andersen et al., 2003; Tomoda et al., 2004; Menon et al., 2007), neointimal hyperplasia (Ai et al., 2018), atherogenesis (Asare et al., 2017), tumorigenesis (Soucy et al., 2010), fatty liver (Zhang X. et al., 2020), obesity (Park et al., 2016), neurodegenerative disorders (Vogl et al., 2015; Zhang L. et al., 2020) and more recently, heart failure (Figure 1). In this review, we first provide an overview of the neddylation pathway and outline the mechanisms by which neddylation modulates protein function; we then focus on heart-specific functions of neddylation. In particular, we will examine the involvement of neddylation in cardiac disease; summarize the role of neddylation and deneddylation in regulating cardiac development, contractility and protein quality control; and identify challenges in understanding the significance of neddylation in health and disease.

## The Neddylation Enzymatic Cascade

First cloned from the murine brain in 1993, NEDD8 is ubiquitously expressed, with the highest expression seen in striated muscles (Kumar et al., 1993; Kamitani et al., 1997). NEDD8 is highly evolutionarily conserved, sharing 100% homology among mouse, rat and human orthologs, and 83% homology with *Arabidopsis* paralogs (Kamitani et al., 1997). NEDD8 is initially synthesized as an 81-amino acid precursor protein that subsequently undergoes proteolytic cleavage to expose glycine-76 at the C-terminus. Several isopeptidases, including UCH-L3, USP21 and NEDP1, have been reported to facilitate NEDD8 maturation (Gan-Erdene et al., 2003; Frickel et al., 2007). Deletion of either UCH-L3 in mice or NEDP1 in cells does not abolish neddylation (Kwon et al., 2004; Bailly et al., 2019), suggesting the functional redundancy of enzymes involved in NEDD8 maturation.

**FIGURE 2:**
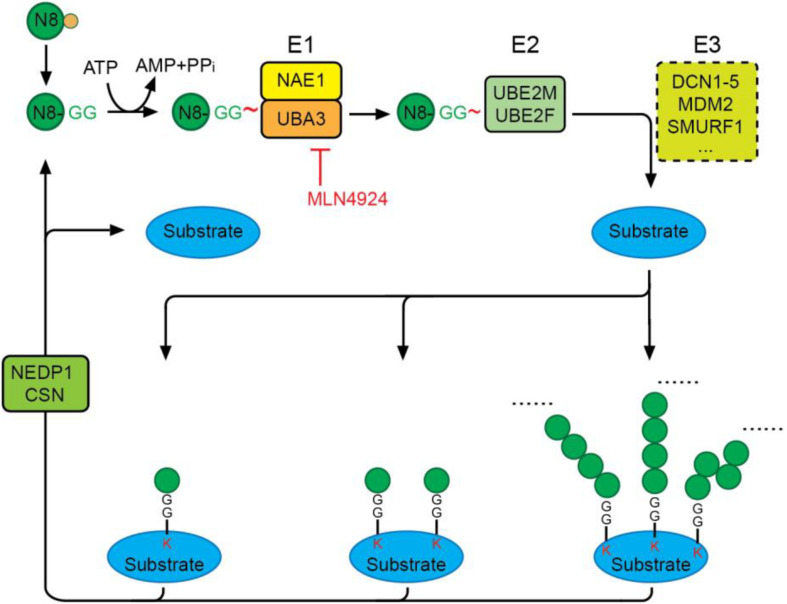
The neddylation cascade. NEDD8 is first synthesized as a precursor that is proteolytically processed to expose its C-terminal glycine. The matured NEDD8 is then activated by forming a thioester linkage with NAE—a heterodimer of UBA3 and NAE1—in an ATP-dependent manner. Activated NEDD8 is then transferred from the active cysteine site of NAE to the active cysteine site of E2 UBE2M or UBE2F. Interaction of E2 with an E3 ligase positions the C-terminal glycine for nucleophilic attack by the lysine residue on the substrate, resulting in covalent linkage of NEDD8 to target proteins via an isopeptide bond. Protein substrates can be modified by mono-, multi-mono, or a chain of NEDD8 via different linkages. The deneddylases, NEDP1 and CSN, catalyze the removal of NEDD8 moieties from substrates. MLN4924 is a specific neddylation inhibitor that acts by preventing NAE-mediated NEDD8 activation.

Conjugation of NEDD8 to proteins is mediated by NEDD8-specific E1-E2-E3 enzymes in a manner similar to ubiquitination (Figure 2). Unlike the highly hierarchical ubiquitination cascade, neddylation is mainly catalyzed by one E1 activating enzyme (NAE), two E2 conjugation enzymes (UBE2M/UBC12 and UBE2F), and a number of E3 ligases. Matured NEDD8 forms a thioester bond with NAE in an ATP-dependent reaction. Once activated, NEDD8 is then transferred between the active cysteine residue of NAE to an E2 conjugating enzyme, either UBE2M or UBE2F. Interaction of the E2 enzyme with an E3 enzyme leads to transfer of the NEDD8 moiety and formation of a covalent isopeptide bond between the C-terminal glycine-76 of NEDD8 and a lysine residue on the substrate protein (Gong and Yeh, 1999; Soucy et al., 2009).

Selective activation of NEDD8, but not other UBLs, is regulated by the NEDD8 E1, NAE. Despite the similarity of NEDD8 and Ub, NAE can distinguish the two protein modifiers at single amino acid resolution by recognizing alanine 72 on NEDD8 (arginine 72 on ubiquitin) (Whitby et al., 1998). NAE is a heterodimer consisting of the regulatory subunit, NAE1, and the catalytic subunit, UBA3, the latter containing an active cysteine residue (C237) essential for the formation of an NAE-NEDD8 thioester. MLN4924 (Pevonedistat), a potent and selective inhibitor of NAE currently in phase I/II/III clinical trials for the treatment of a range of human cancers (https://www.clinicalltrials.gov/), is structurally related to AMP and forms an irreversible covalent NEDD8-MLN4924 adduct. This adduct resembles NEDD8-AMP and blocks the NAE active site, thereby abolishing NEDD8 activation and the NEDD8 pathway. The inhibitory effect of MLN4924 on neddylation is highly specific, and its IC_50_ toward NAE is more than 1000-fold higher than that toward E1s of other UBLs.

Two structurally similar neddylation E2s have been identified in eukaryotic cells: the well-characterized UBC12/UBE2M (Gong and Yeh, 1999) and the less-studied UBE2F (Huang et al., 2009). The two E2s appear to have specificity for different protein substrates, at least with respect to cullin family proteins. For instance, UBC12 pairs with RBX1 to mediate neddylation of CUL1-CUL4, whereas UBC2F interacts with RBX2/SAG to regulate CUL5 neddylation (Kwon et al., 2004). Existing evidence suggests that UBC12 plays a dominant role in neddylation, as overexpression of UBC12 itself is able to induce neddylation, and silencing of UBC12 diminishes neddylation (Gong and Yeh, 1999; Li et al., 2019). Interestingly, a recent study revealed negative regulatory cross-talk between the two E2s such that stress-induced UBC12 acts as a ubiquitin E2 for Parkin that promotes the ubiquitination and degradation of UBE2F (Zhou et al., 2018).

In contrast to the well-defined NEDD8-specific E1 and E2 enzymes, the identities of *bona fide* NEDD8 E3 ligases remain poorly defined. Several research groups have proposed DCN1-5 (defective in cullin neddylation 1-5) as NEDD8 E3 ligases that mediate cullin neddylation, but the exact function of these proteins is controversial. Some groups claim that DCN proteins function independently as NEDD8-specific E3 ligases, whereas others believe that they are simply E3 ligase co-factors (Meyer-Schaller et al., 2009; Huang et al., 2011; Wu et al., 2011). In addition to DCN family proteins, a dozen proteins, including c-Cbl, MDM2, XIAP and SMURF1, have been reported to act as NEDD8 E3 ligases (Xirodimas et al., 2004; Watson et al., 2006; Kim et al., 2008; Broemer et al., 2010; Huang et al., 2011; Embade et al., 2012; Zuo et al., 2013). Interestingly, most of these proteins are also known as Ub E3 ligases. Whether there are specific signals that drive these E3s to promote either neddylation or ubiquitination and whether these E3s work in conjunction with the respective cognate E2 enzymes (either NEDD8 E2 or Ub E2) to determine the fate of the modification remains unclear. Experimental evidence supporting either notion remains scarce.

Like ubiquitination, neddylation is dynamically regulated under normal physiological conditions. Although a number of isopeptidases are capable of deconjugating NEDD8 moieties from protein substrates *in vitro* (Rabut and Peter, 2008; Xirodimas, 2008), only NEDP1 (NEDD8-specific protease 1, also known as SEPN8 and DCN1) and CSN (COP9 signalosome) appear to be NEDD8-specific. CSN is a zinc metalloprotease comprising eight subunits, CSN1-CSN8. CSN primarily removes NEDD8 from cullin family proteins, but also deneddylates other non-cullin proteins (Wei et al., 2008). CSN is indispensable for tissue homeostasis and organismal development, and genetic deletion of any CSN subunit in all tissues and organisms tested causes pathological alterations or lethality (Oron et al., 2002; Lykke-Andersen et al., 2003; Yan et al., 2003; Tomoda et al., 2004; Menon et al., 2007; Zhao et al., 2011). These severe phenotypes are often linked to loss of function of cullin proteins, which are thought to control the stability of approximately 20% of intracellular proteins.

In contrast to CSN, NEDP1, a cysteine protease, appears to be more specific for deconjugation of NEDD8 from non-cullin proteins (Mendoza et al., 2003). Initial studies showed that NEDP1 can remove NEDD8 from both cullins and non-cullin proteins in a cell-free system (Wu et al., 2003). However, deficiency of NEDP1 deficiency in *Arabidopsis*, *Drosophila* or mammalian cells results in substantial accumulation of neddylated proteins without impacting cullin neddylation (Mendoza et al., 2003; Wu et al., 2003; Chan et al., 2008; Vogl et al., 2020), suggesting the specificity of NEDP1 for non-cullin proteins. Interestingly, unlike CSN, NEDP1 is dispensable for the viability of *Drosophila* and *Arabidopsis* (Chan et al., 2008; Mergner et al., 2017), suggesting distinct physiological functions of non-cullin protein neddylation *in vivo*. To date, the physiological function of NEDP-mediated deneddylation in mammals remains unclear.

Similar to the case for ubiquitination and sumoylation, NEDD8 can be conjugated to protein targets in a chain at one or multiple lysine residues. Analyses of neddylated peptides from 609 proteins showed an average of 2.03 neddylation sites per protein, with the majority of NEDD8 substrate having only one or two neddylation sites (Vogl et al., 2020). Proteomics studies have provided direct evidence for polyneddylation (Vogl et al., 2020; Leidecker et al., 2012; Singh et al., 2012), reflecting the fact that the NEDD8 chain can be extended on its K6, K11, K22, K27, K48 and K54 residues. Interestingly, NEDP1 appears to be relatively specific for shortening K6-, K11-, K48- and K54-linked NEDD8 chains because loss of NEDP1 results in accumulation of NEDD8 chains with these linkages, whereas other NEDD8 protease may control the lengths of K22- and K27-linked NEDD8 chains (Vogl et al., 2020). Whether polyneddylation with different linkages differentially impacts protein function has thus far remained unknown.

## Proteome-Wide Identification of NEDD8 Targets

Given the emerging role of neddylation in health and disease, there is a keen interest in identifying NEDD8 substrates at the proteome-wide level. Cullin family proteins, the first and best characterized NEDD8 targets, serve as scaffolds for cullin-RING E3 Ub ligases (CRLs). Cullin family proteins share a conserved neddylation site (IVRIMK^∗^MR), with neddylation serving to stimulate CRL activity. In addition to cullins, a growing list of proteins have been identified as NEDD8 targets. These include Parkin, PINK1, HIF1α, TGFβRII, MDM2, p53, p73, VHL (Wolf et al., 2020), RPS27L, RPS27 (Xiong et al., 2020) and COFILIN (Vogl et al., 2020), among others [also see reviews (Kandala et al., 2014; Enchev et al., 2015)]. Insights gained from characterizations of these new NEDD8 substrates have expanded our understanding of neddylation in diverse cellular pathways. Nevertheless, proteome-wide identification of NEDD8 substrates has been challenging because of the low abundance of neddylated proteins; the transient and reversible nature of the modification; the inability to discriminate among neddylation, ubiquitination and ISGylation; poor efficiency in enriching neddylated proteins using commercially available NEDD8 antibodies; and concerns about the fidelity of targets identified in cells exogenously overexpressing NEDD8 (Hjerpe et al., 2012; Leidecker et al., 2012). Moreover, the promiscuity of neddylation sites on most identified NEDD8 targets prevents designating a *bona fide* NEDD8 substrate and uncovering the functional significance of neddylation.

Several recent studies have attempted to develop new methods to facilitate unbiased screens of NEDD8 targets. The Sutherland group developed a comprehensive platform for analyzing UBL modifications using *in vivo* biotinylation (Pirone et al., 2017). In this modular multi-cistronic expression platform, the expression of Bio/Avi-tagged NEDD8 and biotin ligase BirA^∗^ allows *in vivo* biotinylation of neddylated proteins, and thus efficient enrichment of neddylated proteins, under stringent condition, while the simultaneous introduction of UBE2M may increase the abundance of neddylated proteins. This approach has been applied to identify several UBL modifications in *Drosophila* cells and transgenic flies. Its effectiveness in identifying NEDD8 targets remains to be demonstrated. Other studies have sought to stabilize the neddylated form of NEDD8 targets utilizing a deconjugation-resistant NEDD8 mutant (L73P) or deletion of NEDP1 (Coleman et al., 2017; Keuss et al., 2019). This strategy has identified UBE2M and NSUN2, among other candidates, as novel NEDD8 substrates in HEK293T and Hela cells. To facilitate the isolation and identification of neddylated peptides, a few groups have taken advantage of a monoclonal antibody that specifically recognizes and captures peptides containing lysine residues modified by diglycine (K-ε-GG) (Xu et al., 2010; Coleman et al., 2017; Vogl et al., 2020), an adduct left at ubiquitin-, NEDD8-, and ISG15-modified sites after trypsin digestion. To discriminate neddylation from the other two modifications, the Sheng group generated NEDD8^*R*74*K*^ knock-in HEK293 cells, allowing identification of unique neddylation sites by mass spectrometry after Lys-C digestion and K-ε-GG peptide enrichment. This approach also maintains endogenous NEDD8 levels and thus minimizes potential artifacts resulting from NEDD8 overexpression and non-canonical neddylation mediated by the Ub E1 enzyme, UBE1 (Hjerpe et al., 2012; Leidecker et al., 2012). By screening for differentially expressed neddylated proteins in this NEDD8^*R*74*K*^ knock-in cell line upon MLN4924 treatment and NEDP1 deletion, this study identified 607 neddylation sites on 341 proteins. Gene Ontology enrichment analyses revealed that these NEDD8 substrates belong to diverse protein categories, including DNA/RNA-binding proteins, chaperones, ribosomal proteins, chromatin architecture regulators, Ub- and UBL enzymes, and cytoskeletal proteins, indicating a likely crucial function for neddylation in these cellular processes. Notably, analyses of identified neddylated peptides did not reveal a general consensus sequence for NEDD8 modification, a finding that contrasts with the well-known sumoylation motif (Rodriguez et al., 2001). Despite these advancements, there have not yet been efforts to systematically identify NEDD8 targets *in vivo*. However, a 6xHis-FLAG-*Nedd8* knock-in mouse model has been generated and used to validate neddylation of the mitochondrial proteins, ETFA and ETFB (Zhang X. et al., 2020). This unique mouse line could be a valuable tool for profiling the landscape of endogenous NEDD8 proteins in various tissues in a physiologically and pathologically relevant context.

## Molecular Actions of Neddylation

How neddylation precisely regulates protein function is incompletely understood. Similar to other PTMs, neddylation has an impact on the structure and conformation of target proteins, thus altering the assembly/disassembly of protein complexes as well as protein stability, enzymatic activity, subcellular distribution, and/or binding affinity for DNA and proteins. These mechanisms are often intertwined and can act together to regulate protein function.

### Regulation of the Assembly of Protein Complexes

The cullin family consists of eight members (cullin 1, 2, 3, 4a, 4b, 5, 7, and 9), which serve as scaffolding units for cullin-RING E3 Ub ligases (CRLs). A typical CRL contains one cullin protein, a Ub E2-interacting RING protein (RBX1 or RBX2), an F-box–interacting adaptor, and a substrate-recognizing F-box protein. Evidence collected from biochemical assays has consistently demonstrated the necessity of reversible cullin neddylation for the assembly and activity of CRLs, as extensively reviewed by others (Keuss et al., 2019). Cryo-electron microscopy analyses of the neddylated CRL1^β^ ^*TrCP*^-UBE2D complex and the CSN-CRL2 complex further provide insight into structural mechanisms underlying how NEDD8 activation mediates CRL-catalyzed ubiquitination (Faull et al., 2019; Baek et al., 2020). These analyses show that NEDD8 acts as a nexus for conjugating individual cullins and RBX1/RBX2-activated, ubiquitin-charged E2. Local structural alterations of NEDD8 and CRL domains converge to juxtapose the substrate and ubiquitination active site, leading to ubiquitination of the substrate (Xu et al., 2010). Neddylated CRLs can also form complexes with the deconjugating isopeptidase CSN, and this interaction is required for the NEDD8-dependent activation of CSN. CSN-mediated deneddylation of cullins enables binding of CAND1 to the cullin scaffold and prevents binding of substrate-specific adaptor proteins to the CRL complex, thus effectively sequestering E3 ligase from the intracellular environment (Lydeard et al., 2013). Therefore, dynamic cycling of cullin neddylation/deneddylation is central to the assembly and activity of CRLs.

Another piece of evidence comes from neddylation of SHC (Jin et al., 2013), an adaptor protein that bridges the androgen receptor (AR) to the RAS/ERK pathway. It has been proposed that neddylation of SHC at its N-terminal lysine 3 residue facilitates the formation of a ZAP70-SHC-GRB2 complex that is crucial for downstream ERK activation. Consequent inhibition of neddylation prevents ERK activation and suppresses CD4^+^ T-cell function and airway inflammation.

### Regulation of Protein Stability

A commonly observed consequence of neddylation is a switch in protein stability, with neddylation either stabilizing or destabilizing the substrate. For instance, PPARγ, a crucial player in the regulation of lipid and glucose metabolism, can be modified at unknown lysine sites, which increases its stability by preventing ubiquitination (Park et al., 2016). Similarly, neddylation of other proteins, including the ribosomal proteins RPS27L and RPS27, mitochondrial proteins ETFA and ETFB, and lipogenic transcription factor SREBP1, also increases their stability (Heo et al., 2020; Xiong et al., 2020; Zhang X. et al., 2020).

In contrast, neddylation of the splicing factor SRSF3, which can be induced by treatment with palmitic acid, promotes SRSF3 degradation by the proteasome (Kumar et al., 2019). Mutation of its neddylation sites prevents SRSF degradation and suppresses SRSF3-mediated RNA splicing. Similar effects have also been observed for influenza A viral protein PB2, the transcription factor JunB, and TGFβRII (Oved et al., 2006; Li H. et al., 2016; Zhang et al., 2017).

Several scenarios might explain the distinct impact of neddylation on protein stability. First, Ub and NEDD8 share the same modification sites on substrates and thus must compete for these lysine residues. Second, NEDD8 and Ub may target different lysine on a given substrate. In such cases, neddylation of a substrate on one site might serve as a negative or positive signal for ubiquitination at other sites, possibly by altering their conformation and/or accessibility to their cognate Ub ligases. While experimental evidence needed to support these scenarios is currently lacking, the intimate cross-talk between the two modifications confers additional layers of regulation on protein stability and homeostasis.

### Regulation of Protein Activity

Neddylation has been shown to control the activity of several E3 Ub ligases. For example, neddylation of Parkin, a Ub ligase that mediates the selective degradation of damaged mitochondria through a process known as mitophagy (Ni et al., 2014), was shown to increase Parkin Ub ligase activity in neuronal and cancer cells, possibly owing to its enhanced association with Ub E2 enzymes and substrates (Choo et al., 2012; Um et al., 2012). Interestingly, PINK1, a protein kinase that positively regulates Parkin activity, was also identified as a NEDD8 target, although the functional consequence of PINK1 neddylation was not determined. In a further example, the HECT ubiquitin ligase SMURF1 is also a target of NEDD8 (Xie et al., 2014). SMURF1 is self-neddylated at multiple lysine residues, and its auto-neddylation requires a C426 active site. Similar to the case for Parkin, neddylation of SMURF1 enhances Ub E2 recruitment and thus SMURF1 Ub ligase activity.

NEDD8 can modify protein kinases and influence their enzymatic activity (Wolf et al., 2020). DNA-dependent protein kinase catalytic subunit (DNA-PKcs) is a core component of nuclear DNA-dependent serine/threonine protein kinase (DNA-PK), the latter of which plays an important role in non-homologous end-joining (NHEJ) repair following DNA damage. Neddylation of DNA-PKcs is mediated by the canonical neddylation enzymes, NAE and UBE2M, as well as the E3 ligase, HUWE1 (Guo et al., 2020). Inhibition of DNA-PKcs neddylation with MLN4924 or by silencing HUWE1 impairs DNA-PKcs autophosphorylation without disturbing its stability, leading to diminished NHEJ efficiency.

Neddylation also regulates the activity of several transcription factors, including p53, p73, E2F1 and HIF1α, among others (Xirodimas et al., 2004; Watson et al., 2006; Ryu et al., 2011; Loftus et al., 2012). Notably, neddylation exerts actions independent of its effects on the stability of these targets. For instance, a recent study identified the AR as a novel NEDD8 target (Yu et al., 2020). Mutation of the AR neddylation sites, K475 and K862, increased AR transcription activity, possibly by enhancing the binding affinity of AR for androgen-response elements on its downstream targets. Deletion of NEDD8 in zebrafish was shown to impair ovarian maturation due to hyperactivation of AR, and loss of one copy of AR rescued the phenotype of NEDD8-null zebrafish (Yu et al., 2020).

### Regulation of Subcellular Localization

Several studies have reported that neddylation alters the subcellular distribution of protein targets. Ribosomal proteins are among the most commonly reported neddylation substrates (Xirodimas et al., 2008). For example, neddylation of ribosomal protein L11, mediated by MDM2, prevents L11 translocation from the nucleolus to the nucleoplasm during nucleolar stress (Sundqvist et al., 2009). Nucleolar stress, in turn, induces the rapid and transient recruitment of neddylated L11 to the promoter of p53, thereby enhancing p53 signaling (Mahata et al., 2012). S14, another ribosomal protein, is also modified by NEDD8, and its neddylation is mediated by HDM2 but counteracted by NEDP1 (Zhang et al., 2014). Neddylation appears to cause retention of S14 in the nucleolus and prevent its translocation to the cytoplasm. In addition to ribosomal proteins, the chemokine receptor CXCR5 was reported to be neddylated at lysine 339 in a FANCA-dependent manner (Renaudin et al., 2014). Mutation of this neddylation site diminishes CXCR5 distribution to the cell membrane, suggesting that neddylation is crucial for CXCR5 membrane targeting. In a final example, the E3 ligase RNF111 catalyzes neddylation of the N-terminal lysine of chromatin-localized histone H4. It has been suggested that H4 neddylation may alter chromatin orientation and disrupt internucleosome interactions (Ma et al., 2013), providing access to DNA lesions by DNA damage repair proteins.

## Role of Neddylation in the Heart

The neddylation pathway has consistently proven critical in maintaining various aspects of cellular, organ and organismal function and development. Perturbations in neddylation or deneddylation pathways using either pharmacological or genetic approaches have been shown to cause developmental defects (Lykke-Andersen et al., 2003; Tomoda et al., 2004; Menon et al., 2007), tumorigenesis (Soucy et al., 2010), metabolic disorders (Park et al., 2016), liver dysfunction (Zhang X. et al., 2020), and neurodegenerative disorders (Vogl et al., 2015; Li L. et al., 2016). The importance of neddylation in health and disease is underscored by its role in regulating cell metabolism, proliferation (Xirodimas et al., 2004; Abida et al., 2007; Guihard et al., 2012), cell death (Knorr et al., 2015), autophagy (Luo et al., 2012), cell signaling (Zuo et al., 2013), protein homeostasis (Petroski and Deshaies, 2005; Lipkowitz and Weissman, 2011), gene transcription (Ryu et al., 2011; Loftus et al., 2012; Aoki et al., 2013), and even mitochondrial turnover (Gong and Yeh, 1999; Kwon et al., 2004; Choo et al., 2012; Um et al., 2012).

NEDD8 is one of the most highly expressed UBL proteins in the heart (Zou et al., 2018) and is highly abundant in skeletal and cardiac muscles compared with other organs (Kamitani et al., 1997). Neddylated proteins with a broad range of molecular weights are readily detected in cardiomyocytes under physiological conditions, suggesting that neddylated proteins are more abundant in these cells than in other cell types. In this section, we summarize the emerging roles of neddylation in cardiac development and disease and discuss the mechanisms through which neddylation might control pathways that contribute to disease pathogenesis (Table 1).

**TABLE 1:** Roles of neddylation in the heart.

Targeted components	System modified	Phenotypes	Mechanisms	References
**Association of neddylation with cardiac disease**				
?	Neddylated proteins	Increased in human DCM and ICM	−	Li et al., 2015
?	Neddylated proteins	Increased in mouse DRM	−	Li et al., 2015
**Developing heart**				
αMHC^*Cre*^-NAE1KO	↓neddylation	Perinatal lethality (P2-P3), LVNC	↓CM proliferation, ↓YAP signaling	Zou et al., 2018
MLN4924	↓neddylation	Transient cardiomyopathy	↓CM proliferation	Zou et al., 2019
αMHC^*Cre*^-CSN8KO	↓deneddylation	Premature lethality (∼4 weeks), DCM	↓UPS, ↓autophagy, ↑necroptosis	Su et al., 2011b
**Post-mitotic heart**				
αMHC^*MerCreMer*^-CSN8KO	↓deneddylation	Lethality in 2 weeks, DCM & HF	↓UPS, ↓autophagy, ↑necroptosis	Su et al., 2013
MLN4924	↓neddylation	Exacerbated ISO-induced HF	?	Zou et al., 2019
**Cardiac proteotoxicity**				
Global CSN8 hypomorphism	↓deneddylation	Exacerbated DRM	↓UPS, ↑protein aggregates	Wang et al., 2003
NUB1L O/E	↓atypical neddylation	↓CM damage	↑UPS	Li et al., 2015
NUB1L KD	↑atypical neddylation	↑CM damage	↓UPS	Li et al., 2015

*DCM, dilated cardiomyopathy; ICM, ischemic cardiomyopathy; DRM, desmin-related cardiomyopathy; KO, knockout; LVNC, left ventricle non-compaction cardiomyopathy; UPS, ubiquitin-proteasome system; HF, heart failure; O/E overexpression; KD, knockdown.*

### Association of Neddylation With Cardiac Disease

Dysregulation of neddylation has been implicated in cardiomyopathies of various etiologies. This is evidenced by a significant increase in neddylated proteins in failing hearts from patients suffering from dilated and ischemic cardiomyopathy (Li et al., 2015). Similar findings were also obtained in hearts from mouse models recapitulating human desmin-related cardiomyopathy (Li et al., 2015). The aberrant abundance of neddylation is presumably a consequence of activation of neddylation and/or defects in deneddylation, although experimental evidence for the expression of neddylation enzymes is lacking. Thus far, no polymorphisms or mutations in any neddylation enzymes have been linked to cardiac disease, or in fact any other human disease. Given the essential role of neddylation in maintaining organismal viability, it is likely that loss-of-function mutations of neddylation enzymes are developmentally lethal. However, mutations of neddylation targets have been linked to a number of cardiomyopathies. For instance, CUL3 and LZTR1, an adaptor for the CUL3 Ub ligase complex, have been linked to familial hypertension and Noonan syndrome, respectively (Schumacher et al., 2015; Steklov et al., 2018), both of which exhibit cardiac defects. Moreover, homozygous mutations in KLHL24, another substrate-recognizing adaptor protein for CUL3 Ub ligase, have been shown to cause hypertrophic cardiomyopathy in humans (Hedberg-Oldfors et al., 2019). Knockdown of the KLHL24 homolog in zebrafish also results in ventricular failure, providing additional evidence for KLHL24 as a HCM-associated gene (Hedberg-Oldfors et al., 2019). In line with these findings, clinical investigations of the promising anticancer drug MLN4924 have revealed that cardiac failure is a major adverse event (Shah et al., 2016; Swords et al., 2017). Collectively, these lines of evidence implicate neddylation in cardiac disease, calling for an in-depth investigation of its role in the heart.

### Neddylation in Cardiac Development and Congenital Heart Disease

Heart development is a complex and tightly regulated series of events requiring precise spatiotemporal regulation of various signaling cascades and cell populations to form a functionally competent pumping organ. As is the case for SUMO modifications (Mendler et al., 2016), PTMs with Ub-like proteins may be an important mechanism underlying cardiac maturation and development.

Existing evidence suggests that highly active and balanced neddylation is critical for perinatal cardiac development. Neddylation is developmentally downregulated in the developing heart: both neddylated proteins and neddylation enzymes are highly expressed in embryonic hearts, but are significantly downregulated at 1 week after birth (Zou et al., 2018), a time when cardiomyocytes exit the cell cycle. αMHC^*Cre*^-mediated deletion of the regulatory subunit of the NEDD8 E1 enzyme, NAE1, in mice results in cardiac-specific inhibition of neddylation. Mice lacking NAE1 exhibit cardiomyocyte proliferation arrest as early as embryonic (E) day 14.5 and show pronounced ventricular non-compaction by E16.5, which eventually lead to heart failure and inevitable neonatal lethality (Zou et al., 2018). Moreover, transient inhibition of neddylation by administration of MLN4924 to neonatal rats for 3 days was shown to cause diminished cardiomyocyte proliferation, cardiac hypertrophy and deteriorated cardiac function (Zou et al., 2019), supporting the importance of neddylation in perinatal cardiac growth. Although cardiac function in these animals is restored to a level comparable to their wild-type counterparts in adulthood, they remain more susceptible to isoproterenol-induced heart failure (Zou et al., 2019). This suggests that even transient disruption of neddylation in the neonatal stage can lead to permanent deleterious effects on cardiac function in adulthood.

Interestingly, blocking deneddylation in the developing heart can also be catastrophic. Inhibition of CSN deneddylase activity by cardiac-specific deletion of CSN8 was shown to increase total neddylated proteins in the heart. These CSN8-deficient mice developed cardiac hypertrophy by 2 weeks of age, dilated cardiomyopathy with largely reduced contractility at 3 weeks of the age, and ultimately died of heart failure by 4 weeks of age (Su et al., 2011b). These *in vivo* findings indicate that tight control of the degree of neddylation is essential for normal cardiac development.

Myocardial neddylation not only acts through post-translational pathways but also at the transcriptional level to regulate cardiac development. Studies in cancer cells have consistently demonstrated that neddylation directly regulates the stability of a wide range of cell cycle inhibitors (Soucy et al., 2009), an action that may account for the observed arrest of cardiomyocyte proliferation in NAE1-deficient hearts. In the embryonic heart, neddylation also controls the transcription of numerous cell cycle genes through the Hippo-YAP (Yes-associated protein) pathway, which plays a crucial role in heart morphogenesis by enabling cardiomyocyte proliferation and differentiation (Huang et al., 2005; Wackerhage et al., 2014; Zhou et al., 2015; Wang et al., 2018). Inhibition of neddylation in the heart stabilizes the Hippo kinases MST1 and LATS2, which in turn promotes phosphorylation of YAP in the heart, preventing its nuclear translocation and subsequent transactivation of cell cycle genes (Zou et al., 2018). Mechanistically, CUL7 acts as a Ub ligase for MST1 and promotes its degradation in cardiomyocytes (Zou et al., 2018). Thus, neddylation enables cardiac chamber maturation, at least in part, through temporal inactivation of Hippo kinases during development. Consistent with this, transcriptomic analyses of CSN8-deficient hearts have revealed dysregulated expression of a wide array of genes involved in diverse pathways (Abdullah et al., 2017). The role of neddylation in the control of gene expression patterns is also in agreement with studies in flies (Oron et al., 2007; Ullah et al., 2007), which suggest that CSN4 acts as a transcription repressor during embryonic development and controls chromatin remodeling, either directly or indirectly.

The immediate downstream effectors of neddylation in cardiac development could be linked, at least in part, to cullin proteins. In addition to CUL7, mentioned above, CUL3 is also critical for perinatal cardiac development. Mice with a CUL3 deficiency quickly develop heart failure after birth and die within 1 week (Papizan et al., 2018). The underlying mechanisms were purported to be dysregulated cardiac proteome and metabolism, although whether such alterations are primary or secondary to cardiac failure remains unclear. Additionally, the CUL5-Asb2α-Filamin A axis has been implicated in heart morphogenesis (Métais et al., 2018). Asb2α is an adaptor protein of CUL5 Ub ligase that promotes the degradation of the cytoskeleton protein filamin A. Global deletion of Asb2α in mice causes cardiac malformations with defects in valve, atrium and ventricular development before E12.5 and lethality thereafter (Métais et al., 2018), phenotypes that are largely recapitulated in mice with conditional deletion of the same gene in cardiomyocytes. These observations suggest that further efforts to determine the role of cullins in cardiac development are warranted.

### Neddylation and Dilated Cardiomyopathy and Heart Failure

Although the biological function of neddylation in developmental processes has been extensively studied and interpreted in the context of cell differentiation and division (Enchev et al., 2015), a number of studies have started to unveil the functional importance of neddylation in post-mitotic organs, including the heart (Su et al., 2011a,b, 2013; Vogl et al., 2015). As noted above, mice lacking CSN8 develop dilated cardiomyopathy and heart failure at the age of 3 to 4 weeks. Since deletion of CSN8 does not cause destabilization of the CSN complex until the first week after birth, when cardiomyocytes have almost completed the last round of proliferation (Su et al., 2011b), the severe cardiac phenotype is not likely attributable to defects in cardiomyocyte proliferation but rather underscores a pivotal role for deneddylation in post-mitotic cardiomyocytes. Indeed, tamoxifen-induced knockout of CSN8 in the adult heart led to the rapid development of heart failure and premature death within 2 weeks after induction (Su et al., 2013). These findings demonstrate the necessity of CSN-mediated deneddylation in the maintenance of cardiac integrity in postnatal and adult hearts. Whether direct modulation of neddylation, for instance, through deletion of the E1 subunits, NAE1 and UBA3, has an impact on adult heart function remains to be explored.

Neddylation appears to be crucial for cardiomyocyte survival in the post-mitotic heart. While inhibition of neddylation has been shown to induce apoptosis in cancer cells (Soucy et al., 2009), this effect seems to be cell-type specific, because neither deletion of NAE1 in embryonic hearts nor knockout of CSN8 in postnatal hearts results in prevalent cardiomyocyte apoptosis. Instead, loss of CSN8 in postnatal and adult hearts induces massive cardiomyocyte necroptosis (Su et al., 2011b, 2013), a form of programed necrotic cell death mediated by the RIPK1-RIPK3 pathway (Del Re et al., 2019). RIPK1, RIPK3, MLKL and protein carbonyls were found to be upregulated in CSN8-deficient heart in association with inhibition of the cleavage/activation of caspase 8 (Xiao et al., 2020). All of these findings are consistent with activation of the RIPK1-RIPK3 pathway. Moreover, both administration of the RIP1K inhibitor necrostatin-1 and deletion of one allele of RIPK3 attenuated cardiomyocyte cell death and increased the lifespan of CSN8-deficient mice (Xiao et al., 2020). The upstream event that triggers necroptosis in CSN8-deficient hearts may be related to dysregulated Ca^2+^ dynamics. Voltage-gated Ca^2+^ channels mediate Ca^2+^ influx into cells and regulate muscle contraction and other intracellular signaling events. Excessive Ca^2+^ influx has been shown to induce necrosis or apoptosis, depending on the energetic status of the cell (Orrenius et al., 2003). Interestingly, a previous study revealed an interaction between CSN5 and the α1c subunit of the L-type Ca^2+^ channel, and speculated that this interaction inhibits channel activity (Kameda et al., 2006). Thus, a plausible model is that loss of CSN8 triggers Ca^2+^ overload, which in turn activates RIPK1 and RIPK3 leading to necroptosis. Additional experimental evidence is needed to support this hypothesis.

### Neddylation and Cardiac Proteotoxicity

Deregulation of protein homeostasis is considered to be a key pathogenic factor in various cardiac diseases (Su and Wang, 2010; Wang and Robbins, 2014). In addition to directly modulating the functionality of protein substrates, neddylation also regulates the proper function of two interconnected proteolytic pathways: the ubiquitin proteasome system (UPS) and autophagy. Direct evidence for this comes from a study that probed the impact of CSN8 hypomorphism on cardiac proteinopathy (Su et al., 2015), a form of cardiomyopathy that manifests as abundant misfolded proteins and protein aggregates in the myocardium (McLendon and Robbins, 2011). Mice with cardiac overexpression of the misfolded proteins, mutant crystallin B (CryAB^*R*120*G*^) and mutant desmin (D7-Desmin), exhibit cardiac dysfunction, heart failure and premature lethality (Wang et al., 2003), and thus represent a bona fide mouse model of cardiac proteinopathy. Despite having no impact on cardiac function under basal conditions, CSN8 hypomorphism was shown to aggravate CryAB^*R*120*G*^-induced restrictive cardiomyopathy and shorten the lifespan of CryAB^*R*120*G*^ mice (Su et al., 2015). The cardiac phenotype was associated with augmented accumulation of protein aggregates, increased neddylated proteins, and reduced total ubiquitinated proteins. These findings collectively suggest a crucial role for CSN-mediated deneddylation in antagonizing proteotoxic stress in cardiomyocytes.

Because the structure of CSN is similar to that of the 19S proteasome lid, and since CSN is capable of controlling CRLs activity, CSN has long been proposed as a UPS regulator (Li and Deng, 2003). In support of this idea, ubiquitinated and oxidized proteins are elevated in CSN8-deficient mouse hearts together with increased protein aggregates (Su et al., 2011b, 2013). Chaperone proteins, such as Hsp25, Hsp90, and α-crystallin B, are also upregulated in these mutant hearts, likely as an adaptive response to the prevalent proteotoxic stress (Su et al., 2011b). Moreover, both CSN8 deficiency and hypomorphism lead to significant accumulation of a UPS functional reporter in mouse hearts (Su et al., 2011b, 2013, 2015), indicating compromised UPS function. Given the well-established role of neddylation in regulating CRL activity, uncoupling of ubiquitination with subsequent degradation by the proteasome may underlie the deficits in UPS function in the CSN8-deficient myocardium.

Neddylation has been shown to impact several steps in autophagy. On the one hand, autophagy initiation appears to be repressed by neddylation, as evidenced by the fact that inhibition of neddylation in cancer cells causes accumulation of DEPTOR, which inhibits mTOR signaling and leads to autophagy induction (Luo et al., 2012). On the other hand, CSN-mediated deneddylation is required for autophagosome maturation (Su et al., 2011a). CSN8 deficiency impairs autophagy flux and results in the accumulation of autophagosomes. Downregulation of RAB7, a critical factor for vesicle trafficking, may account for observed defects in autophagosome fusion with lysosomes.

Neddylation may regulate proteolysis via mechanisms independent of canonical neddylation enzymes. Under various stress conditions, NEDD8 can be incorporated into an existing ubiquitin chain and form mixed Ub- and NEDD8-modified conjugates (Hjerpe et al., 2012; Leidecker et al., 2012). The presence of hybrid Ub/NEDD8 chains has been further confirmed by proteomics studies (Singh et al., 2012; Vogl et al., 2015). Surprisingly, the formation of mixed Ub and NEDD8 chains requires the Ub E1 enzyme UBE1 but not the neddylation E1 enzyme, and therefore is termed atypical neddylation (Hjerpe et al., 2012; Leidecker et al., 2012). The biological consequences of mixed Ub/NEDD8 modifications remain mysterious. It has been proposed that atypical neddylation prevents excessive Ub chain extension and maintains a free Ub pool that otherwise would be toxic to cells (Hjerpe et al., 2012; Leidecker et al., 2012). These hybrid NEDD8/Ub conjugates may also be less efficiently processed by the proteasome and are prone to form protein aggregates in the nucleus (Maghames et al., 2018), which may protect the nuclear UPS from stress-induced dysfunction.

NUB1L (NEDD8 ultimate buster 1 long), a protein that was originally found to decrease neddylated proteins (Kito et al., 2001), has been shown to enhance UPS function in cardiomyocytes (Li et al., 2015). Notably, NUB1L can also bind to atypically neddylated proteins. Ectopic expression of NUB1L in cardiomyocytes reduces mixed Ub- and NEDD8-modified proteins, enhances degradation of a UPS functional reporter and misfolded CryAB^*R*120*G*^ protein, and attenuates cellular damage in response to proteotoxic stress, whereas depletion of NUB1L does the opposite (Li et al., 2015). Structurally, NUB1L contains a UBA (Ub-associating domain) domain and a UBL domain and thus belongs to the UBA-UBL protein family. Members of this family often support proteasomal activity by shuttling ubiquitinated proteins to the proteasome for degradation. Indeed, targeting ubiquilin 1, another member of the UBA-UBL family, in mouse hearts has demonstrated the essential role of this protein in coupling cardiac ubiquitination to the proteasome and limiting myocardial ischemia-reperfusion injury (Hu et al., 2018). Future studies are warranted to determine whether NUB1L acts in a similar way to facilitate the removal of mixed Ub- and NEDD8-modified proteins in the myocardium.

## Future Perspectives

Several key findings in the last decade have revealed an important role of the NEDD8 system in cardiac development and function under physiological and pathological conditions. Current evidence suggests that a tight balance between neddylation and deneddylation is essential for the maintenance of normal heart physiology. This regulatory dynamic mainly occurs through regulation of cardiomyocyte proliferation, maturation, survival, and proteolysis and reflects modification of a diverse cardiac proteome that remains incompletely characterized. Further elucidation of the pathophysiological significance of neddylation in the heart, as well as delineation of the underlying mechanisms, await answers to a number of key questions.

First, does neddylation play any role in early cardiogenic events? Cardiogenesis begins as early as E7.5 in mice and involves the coordination of diverse cardiac resident cells, including atrial and ventricular cardiomyocytes, endothelial cells, smooth muscle cells, pro/epicardial cells, valvular components, and Purkinje cells. NAE1-floxed and UBA3-floxed mouse models are readily available for dissecting the role of neddylation in individual cell lineages and determining their contribution to cardiac development. Also important is the advent of human induced pluripotent stem cell (iPSC) technology, which has generated significant enthusiasm for its potential application in basic cardiac research. By genetically or pharmacologically modulating neddylation in iPSC-derived cardiomyocytes, it may be possible to mechanistically define whether and how neddylation controls cardiomyocyte differentiation and maturation. Findings from such studies may provide new insights into how post-translational mechanisms precisely control cardiogenesis.

Second, neddylation is developmentally downregulated, raising the question of whether it is dispensable for cardiac homeostasis in adulthood. As discussed above, the importance of neddylation in terminally differentiated, post-mitotic/quiescent cells has not yet been established. In the case of neurons, another cell type long purported to lose proliferative capacity soon after birth, inhibition of neddylation results in synaptic spine loss, decreased synaptic activity and cognitive deficits, supporting the conclusion that neddylation likely broadly regulates cellular processes beyond its specific effects on cell division. The cardiotoxicity of the neddylation inhibitor MLN4924 (Shah et al., 2016; Swords et al., 2017) underscores the importance of understanding underlying mechanisms in developing therapeutic options targeting neddylation. Moreover, patients with preexisting cardiovascular disorders, who were excluded from these clinical studies, could be more sensitive to MLN4924-induced cardiotoxicity. Thus, it is also essential that the impact of neddylation on cardiac remodeling be examined in hearts challenged by different pathological insults.

Third, is the neddylation of cullin proteins critical for cardiac development and function? Whether cullin family proteins—the best-characterized NEDD8 targets—are critical regulators of cardiac function has yet to be determined. While *in vivo* studies using animal models deficient for a given cullin have demonstrated the importance of individual cullins in organ and organismal development (Sarikas et al., 2011), the significance of cullin neddylation has not been determined *in vivo*. Generation and characterization of cullin neddylation-deficient mutants will provide proof-of-principal evidence for the functional importance of cullin neddylation.

Fourth, does non-cullin neddylation play any role in the heart? To date, the biological function of neddylation has been interpreted primarily based on its effects on cullin family proteins—important targets of neddylation. Yet there are many other myocardial proteins that are modified by NEDD8. Despite substantial increases in neddylated proteins, including non-cullin proteins, in human and mouse failing hearts (Li et al., 2015), it remains unclear whether the accumulation of non-cullin neddylated proteins is pathogenic or adaptive to the cardiac remodeling process. NEDP1 modulates non-cullin deneddylation (Ehrentraut et al., 2013; Mergner et al., 2015). Thus, targeting the deneddylase NEDP1 could be a feasible strategy for probing the importance of non-cullin neddylation in the heart.

Finally, which proteins are modified by NEDD8 in cardiomyocytes? Thus far, proteomics studies that have profiled the NEDD8 proteome have mainly been conducted using cultured cell lines. However, it is conceivable that the NEDD8 proteome is cell-type specific and varies under different developmental and disease conditions. Thus, it is crucial that physiologically relevant NEDD8 substrates be identified in a cell type- and modification site-specific manner. To this end, inhibition of deneddylation through deletion of either CSN or NEDP1 using a Cre-LoxP strategy may offer an exciting approach for inducing accumulation of neddylated proteins in specific cell types/organs *in vivo*. This could be coupled with enrichment of NEDD8-modified peptides using a K-GG antibody, thereby improving the efficiency and fidelity of the identification. Nevertheless, it is important to bear in mind that the K-GG antibody cannot distinguish peptides generated from Ub-, NEDD8- and ISG15-modified proteins. To address this limitation, researchers have recently developed an antibody (UbiSite) that specifically recognizes the unique ubiquitin remnant on protein substrates after endopeptidase LysC digestion (Akimov et al., 2018). Development of an antibody specific for a unique NEDD8 remnant (K-ε-ILGGSVLHLVLALRGG) following Lys-C digestion could be another option for improving the effectiveness of NEDD8 target identification (Jeram et al., 2010). Thus, identification of the NEDD8 proteome is possible and could provide invaluable new insights into the biological functions of neddylation, an existing and yet unsolved mystery.

## Author Contributions

JLi, JZ, and RL drafted the manuscript. JLiu and HS revised the manuscript. All authors contributed to the article and approved the submitted version.

## Conflict of Interest

The authors declare that the research was conducted in the absence of any commercial or financial relationships that could be construed as a potential conflict of interest.
